# Effect of Chronic Exposure to Textile Wastewater Treatment Plant Effluents on Growth Performance, Oxidative Stress, and Intestinal Microbiota in Adult Zebrafish (*Danio rerio*)

**DOI:** 10.3389/fmicb.2021.782611

**Published:** 2021-11-25

**Authors:** Chun Wang, Zixi Yuan, Yingxue Sun, Xiaolong Yao, Ruixuan Li, Shuangshuang Li

**Affiliations:** ^1^School of Ecology and Environment, Beijing Technology and Business University, Beijing, China; ^2^State Environmental Protection Key Laboratory of Food Chain Pollution Control, Beijing Technology and Business University, Beijing, China; ^3^College of Energy and Environmental Engineering, Hebei University of Engineering, Handan, China

**Keywords:** textile effluent, *Danio rerio*, chronic toxicity, antioxidant, inflammatory response, intestinal microbiota

## Abstract

The ever-increasing production and processing of textiles will lead to greater risks of releasing pollutants into the environment. Textile wastewater treatment plants (TWTPs) effluent are an important source of persistent toxic pollutants in receiving water bodies. The effects of specific pollutants on organisms are usually studied under laboratory conditions, and therefore, comprehensive results are not obtained regarding the chronic combined effects of pollutants under aquatic environmental conditions. Thus, this study aimed to determine the combined effects of TWTP effluents on the growth performance, oxidative stress, inflammatory response, and intestinal microbiota of adult zebrafish (*Danio rerio*). Exposure to TWTP effluents significantly inhibited growth, exacerbated the condition factor, and increased the mortality of adult zebrafish. Moreover, markedly decreases were observed in the activities of antioxidant enzymes, such as CAT, GSH, GSH-Px, MDA, SOD, and T-AOC, mostly in the intestine and muscle tissues of zebrafish after 1 and 4 months of exposure. In addition, the results demonstrated that TWTP effluent exposure affected the intestinal microbial community composition and decreased community diversity. Slight changes were found in the relative abundance of probiotic *Lactobacillus*, *Akkermansia*, and *Lactococcus* in zebrafish guts after chronic TWTP effluent exposure. The chronic toxic effects of slight increases in opportunistic pathogens, such as *Mycoplasma*, *Stenotrophomonas*, and *Vibrio*, deserve further attention. Our results reveal that TWTP effluent exposure poses potential health risks to aquatic organisms through growth inhibition, oxidative stress impairment of the intestine and muscles, and intestinal microbial community alterations.

## Highlights

- The chronic combined biotoxicity of zebrafish exposed to TWTP effluents was studied.
- TWTP effluents exposure significantly inhibited growth and increased mortality.
- TWTP effluents exposure increased MDA and decreased antioxidant enzyme activity.
- TWTP effluent affected the intestinal microbiota and decreased community diversity.

## Introduction

The textile industry is one of the most complicated industries of the manufacturing sector. China is the largest producer of dyed and finished products in the world. The rapid development of the textile industry is leading to the production of large amounts of textile wastewater (Zhou et al., 2020; Li F. et al., 2021). However, textile wastewater contains many harmful biodegraded and toxic substances. It is one of the types of industrial organic wastewaters that are highly difficult to purify (Kishor et al., 2021).

The composition of textile wastewater is extremely complex, particularly owing to the use of new PVA sizes, auxiliaries, and other organic compounds that are resistant to photolysis, oxidation, and biodegradation (Holkar et al., 2016; Paździor et al., 2018; Chowdhury et al., 2020). Therefore, it has become increasingly difficult to purify textile wastewater (Kuleyin et al., 2021; Wang T.Z. et al., 2021). Strict standards in the form of “Discharge standards of water pollutants for dyeing and finishing of the textile industry (GB 4287-2012)” and “Integrated wastewater discharge standard (GB 8978-1996)” were laid out for controlling textile effluent discharge. However, owing to the complex chemical characteristics of dyeing and finishing wastewater, the existing treatment processes of combined chemical coagulation, electrochemical oxidation, and activated sludge treatment cannot completely remove all types of pollutants therein (Li et al., 2014; Zou et al., 2017; Han T.X. et al., 2021; Rodríguez-Narváez et al., 2021). Known pollutants (at relatively low concentrations) and unknown complex pollutants persist in the effluents of textile wastewater treatment plants (TWTPs) even if these meet the discharge standards (Xue et al., 2019; Yuan et al., 2020; Zafar et al., 2021). The chronic ecological toxicity caused to aquatic organisms in receiving waters by long-term exposure to such combined pollutants cannot be insignificant.

Several studies have been performed on ecotoxicology of specific substances (single or combined), such as heavy metals with high biological toxicity occurring in dyeing and finishing wastewater. Toxicological studies of pure substances are especially frequent (Giorgetti et al., 2011; Liang et al., 2017; Khan et al., 2019; Chaturvedi et al., 2021). Previous studies have indicated that dyes, heavy metals, and flame retardants can cause different types of toxicity, such as hepatotoxicity, hematotoxicity, and neurotoxicity, in many aquatic species (Abe et al., 2018; Chen X.P. et al., 2021; Renu et al., 2021; Xia et al., 2021). The effects of toxicants in textile wastewater are generally mediated either by the induction of intracellular oxidative stress and lipid peroxidation or through bioaccumulation in tissues of the affected organisms (Dreier et al., 2021; Kumar et al., 2021). However, there is paucity of research on physiological oxidative stress caused by long-term exposure to contaminants in TWTP effluents.

The intestine is one of the first barriers to pollutant entry in fish. It is considered the primary site for digestion, absorption, transportation of nutrients, and toxin exposure, due to its broad surface areas and physiological characteristics (Anvarifar et al., 2018; Jia et al., 2021). Thus, complete intestinal structure and function are essential to the health of the host. Intestinal functions are strongly influenced by the associated microbial community, which gets affected by toxic pollutants (antibiotics, heavy metals, etc.) when exposed to TWTP effluents (Evariste et al., 2019; Licht and Bahl, 2019; Duan et al., 2020; Jia et al., 2021). However, there is inadequate information on the changes in intestinal health of fish after TWTP effluent exposure.

Aquatic organisms are exposed to pollutants through ingestion of TWTP effluents, which may alter the microbiota composition in their intestines (Licht and Bahl, 2019). Dysbiosis of the gut microbiome may lead to the onset of various diseases in host organisms (Duan et al., 2020; Kayani et al., 2021). The correlation between several diseases and changes in the composition of intestinal microbiota has been reported (Bajaj and Khoruts, 2020; Sun et al., 2021). Numerous factors, such as developmental stage, environmental factors (e.g., water salinity and temperature), geographic habitats, and species, can modulate the composition of gut microbiota (Wang A.R. et al., 2018; Zhao et al., 2020; Jia et al., 2021; Morris et al., 2021; Zhang et al., 2021). However, information on the effects of TWTP effluents on the gut microbiota of aquatic organisms is limited.

Zebrafish (*Danio rerio*) has been widely used in toxicological studies of specific pollutants and can serve as a bio-indicator for assessing the risks of environmental pollution in TWTP effluents. To understand the effect of TWTP effluent exposure on intestinal health and microbiome in zebrafish, the survival rate, physical parameters, and levels of catalase (CAT), glutathione (GSH), glutathione peroxidase (GSH-Px), malondialdehyde (MDA), superoxide dismutase (SOD), total antioxidant capacity (T-AOC), and the composition and diversity of intestinal microbiota were investigated in zebrafish. This study sheds light on the environmental risks of TWTP effluents in aquatic environments.

## Materials and Methods

### Zebrafish Culture

Adult male zebrafish (*Danio rerio*) at 3 months of age were purchased from the China Zebrafish Resource Center and were acclimatized for 2 weeks before the experiment. The acclimatized fish were cultured in a semi-static system containing charcoal-filtered, fully aerated tap water, and continuous aeration. Water parameters were as follows: temperature of 26 ± 1.0°C and pH 7.5 ± 0.5. The fish were fed twice daily at 08:30 and 17:30 with newly hatched *Artemia salina* with apparent satiety. The photoperiod cycle was 14 h:10 h (light:dark). All protocols conformed to the National Institutes of Health Guide for the Care and Use of Laboratory Animals. All experimental procedures were performed strictly according to the guidelines for the care and use of experimental animals (Olfert et al., 1993).

### Experimental Design

After 2 weeks of acclimation, 120 healthy adult male zebrafish (Average weight 0.18 ± 0.06 g) were randomly divided into two groups including six tanks. Experiments on the TWTP effluent-exposed group and control group with aerated tap water were performed in triplicate per treatment, with 20 fish per replicate, under the culture conditions described above. The TWTP effluent was sampled from the discharge outlet of three typical textile wastewater treatment plant in Shengze town (Suzhou, China), which was one of the biggest textile industry industrial park in China. The effluents containing all components of textile wastewater contaminants were well-mixed with equal proportions and used for exposure experiment. During the 4 months of the experimental period, the fish in the control and treatment groups were fed diets prepared in the same way as during the acclimatization period.

### Morphological Analysis

After a 4-month chronic exposure experiment, all fish were anesthetized with 0.03% tricaine (MS-222; Sigma, United States) (Dong et al., 2020). The body length and body weight of each individual in each group were measured and recorded (*n* = 60 per group). After being disinfected with 70% alcohol, the fish were dissected under sterile conditions, and the liver was weighed. The condition factor (K-factor) and hepatosomatic index (HSI) were determined using the following formulas:


K-factor=weight/length×3100,

HSI=liverweight/bodyweight×100.

Cumulative mortality rates were also determined in the experiment.

### Sampling Collection

After 1 month and at the end of feeding trial, zebrafish were deprived of food for 24 h before sample collection. Aseptic forceps and dissecting scissors were used to dissect the anesthetic fish, including gut tissues, gut contents, and muscles, on a clean bench. For biochemical analysis, fish muscles and gut tissues from eight fish per tank were dissected, washed with sterile physiological saline, and mixed as one sample. After 1 month, the tissue samples were collected and snap-frozen in a liquid nitrogen tank for subsequent analysis. At the end of the experiment (i.e., after 4 months), gut contents and water were collected in addition to the tissue samples above. Samples of intestinal contents were aseptically scraped with a sterile scalpel and were collected into sterile tubes (Eppendorf, Germany) for subsequent microbial DNA extraction. Water samples from six tanks were collected with a sterile 1 L beaker and then sequentially filtered through 1.2 μm filter paper (Whatman, NJ, United States) and 0.22 μm filter paper (Millipore, MA, United States) to collect the bacteria. All samples were stored at −80°C until analysis.

### Biochemical Analysis

All tissue samples were homogenized (1:9, wt/vol) with ice-cold physiological saline and centrifuged at 3,000 rpm for 10 min. Several crucial oxidation resistance and immunological parameters, including superoxide dismutase (SOD), glutathione peroxidase (GSH-Px), glutathione (GSH), catalase (CAT), malondialdehyde (MDA), and total antioxidant capacity (T-AOC) were quantified using commercial kits according to the manufacturer’s instructions (Jiancheng Bioengineering Institute, Nanjing, China).

### DNA Extraction and PCR Amplification

Microbial genomic DNA was extracted from the gut contents samples using the PowerFecal ^®^ DNA Isolation Kit (Mo Bio, CA, United States) following the manufacturer’s instructions. Total DNA was extracted from the water samples using PowerWater ^®^ DNA Isolation Kit (Mo Bio, CA, United States). DNA from the two filters (1.2-μm and 0.22-μm) were pooled together as one sample. The extracted DNA was quantified by spectrophotometric analysis (Nanodrop ND-1000, Thermo Fisher) and agarose gel electrophoresis (1.0%, w/v). Finally, gut and water DNA were stored at −20°C until further analysis.

The V4-V5 hypervariable region of the bacterial 16S rRNA gene was amplified with a forward primer 515F and a reverse primer 909R (Supplementary Table 1) containing a unique 12-base barcode (Ye et al., 2014). The PCR mixture (25 ml) contained: 1 × PCR buffer, 1.0 mol/L each primer, 1.5 mmol/L MgCl_2_, each dNTPs at 0.4 mol/L, 0.5 U of Ex Taq and 10 ng genomic DNA. The amplification thermal cycling consisted of 94°C for 3 min; 30 cycles of 94°C for 40 s, 56°C for 60 s, and 72°C for 60 s; and 72°C for 10 min. Each sample was subjected to triplicate reactions to minimize PCR bias, and these were finally combined. DNA products were visualized using 1.4% agarose gel electrophoresis and purified using the AxyPrep DNA Gel Extraction Kit (Axygen Biosciences, Union City, CA, United States).

### MiSeq Sequencing and Data Analysis

DNA was purified and quantified. All the samples were combined according to the quantification results. High-throughput sequencing was conducted using an Illumina MiSeq system, as described previously (Wang C. et al., 2018).

Raw sequencing data were processed using the QIIME pipeline (Caporaso et al., 2010). The paired-end reads were merged using the FLASH-1.2.8 (Magoč and Salzberg, 2011) and assigned to each sample for further analysis. UCHIME was used to identify and remove chimeric sequences (Edgar et al., 2011). An operational taxonomic unit (OTU) was generated using a 97% identity threshold. Each sample was rarefied to the same sequencing depth.

Alpha diversity was estimated by Chao1, Shannon–Wiener, Simpson’s diversity, and Good’s coverage indices, which were applied to describe the species composition in one specific treatment and the differences among treatments (Myer et al., 2016). Beta diversity was assessed using unweighted UniFrac-based Principal Coordinates Analysis (PCoA). Taxonomy was assigned using the RDP classifier (Cole et al., 2003).

### Statistical Analysis

Data expressed as mean ± SD were determined using Microsoft Excel 2016 (Microsoft Office 2016, Microsoft, United States), and all data were subjected to statistical analysis using SPSS (version 13.0; SPSS Inc., Chicago, IL, United States). Differences between treatments were evaluated by one-way analysis of variance (ANOVA) followed by Duncan’s multiple range test. Before ANOVA, all valid data were normal distribution and conducted homogeneity of variance test. Significant differences were set at *P* < 0.05.

## Results and Discussion

### Growth and Condition Factor in Adult Zebrafish

Supplementary Table 2 shows the growth and condition factors of adult zebrafish after exposure to TWTP effluents. After 4 months of exposure, the mean weights and lengths of zebrafish were 0.35 ± 0.03 and 0.32 ± 0.03 g, 3.44 ± 0.12 and 3.20 ± 0.18 g for control and treatment group, respectively. A significant difference in growth was observed between the control and treatment groups (*P* < 0.01). Significant differences in K-factor (*P* < 0.01) and HIS (*P* < 0.05) were also found. The effects of TWTP effluents on the survival of zebrafish are shown in Supplementary Figure 1. The mortality of zebrafish with chronic exposure to TWTP effluents was much higher than that in aerated tap water. These results indicate that TWTP effluents inhibit the growth of zebrafish.

Growth and condition factors (K-factor calculated as weight/length^3^ × 100) are important indicators commonly used to evaluate health status of zebrafish and the effects of pollutants on these. In this study, compared with that in the control group, chronic TWTP effluents exposure affected the body length, weight, and K-factor of zebrafish, also causing chronic developmental toxicity that adversely affected growth performance. Similar findings were reported in another study, in which persistent pollutants in effluents from the textile dyeing industry caused growth inhibition (Methneni et al., 2021).

The HSI (calculated as liver weight/body weight × 100) is often used to evaluate liver growth and liver function in fish under dietary exposure to chemicals or diet regulation (Hamid et al., 2021; Zhao et al., 2021). In this study, HSI decreased significantly in the treatment group (*P* < 0.05, Supplementary Table 2). A significant effect on the HSI value was also reported when zebrafish were exposed to pharmaceutical and personal care products (Hamid et al., 2021).

Hepatosomatic index is considered to be an important indicator of the effects of toxic substances on liver development and metabolism (Field et al., 2008). The significant decrease in HSI in the treatment group might be explained by the decrease in lipids synthesized in the liver. It has been shown that liver weight increases with more hepatic lipids (Hussain et al., 2017). Eventually the zebrafish liver atrophies and the detoxification capacity of the liver is reduced. Furthermore, the zebrafish may be more susceptible to infestation by toxic substances in the effluent, causing a variety of diseases. Similarly, it has been reported that significantly decreased HIS and liver lesions were found by environment-related pollutants exposure, which may also lead to the abnormality of liver development and metabolism (Deng et al., 2010).

In this study spanning 4 months, mortality was higher in the effluent-exposed group compared to the control. Controversial results had been found in previous reports (Han Y. et al., 2021; Rothe et al., 2021). Effects of chronic toxicity on survival rate was showed in this study.

### Oxidative Stress of Intestine and Muscle

**FIGURE 1:**
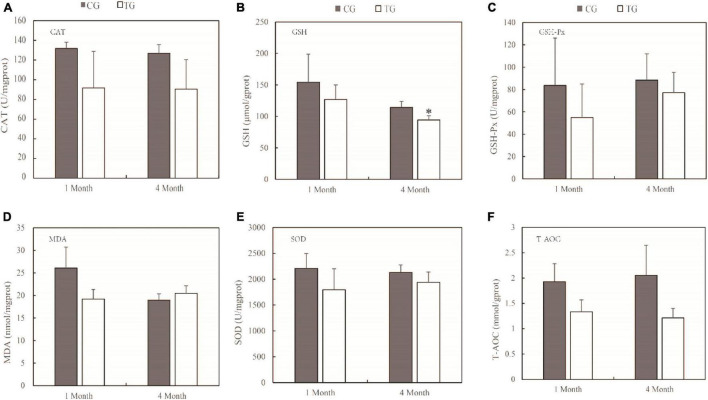
Antioxidant responses of adult zebrafish exposure to TWTP effluents (1 and 4 months) in the gut tissue. Catalase (CAT) **(A)**, glutathione (GSH) **(B)**, Glutathione peroxidase (GSH-Px) **(C)**, malondialdehyde (MDA) **(D)**, superoxide dismutase (SOD) **(E)**, and total antioxidant capacity (T-AOC) **(F)**. Data are presented as the mean ± SD (*n* = 24 fish/treatment). *Stands for significant differences among treatments compared with the control using one-way analysis of variance (ANOVA) followed by Duncan’s multiple comparisons (*P* < 0.05).

**FIGURE 2:**
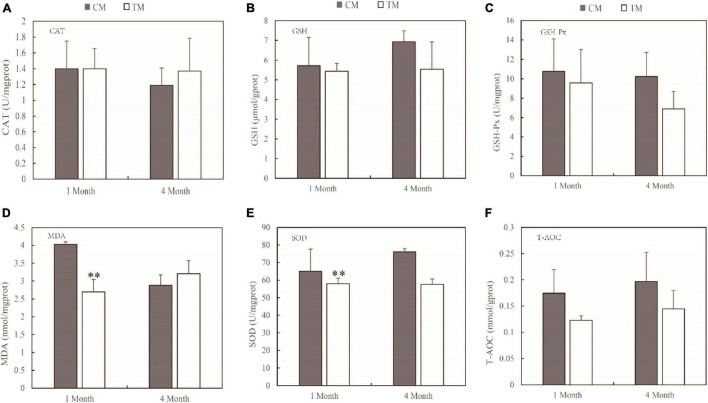
Antioxidant responses of adult zebrafish exposure to TWTP effluents (1 and 4 months) in the muscle tissue. Catalase (CAT) **(A)**, glutathione (GSH) **(B)**, Glutathione peroxidase (GSH-Px) **(C)**, malondialdehyde (MDA) **(D)**, superoxide dismutase (SOD) **(E)**, and total antioxidant capacity (T-AOC) **(F)**. Values presented are mean ± SD (*n* = 24 fish/treatment). ^**^Stands for extremely significant differences among treatments compared with the control (*P* < 0.01).

The primary mechanism of TWTP effluent-induced chronic toxicity is oxidative stress. The effects of TWTP effluents on intestinal and muscle oxidative stress are shown in Figures 1, 2, respectively. The antioxidant activities of catalase (CAT), glutathione (GSH), glutathione peroxidase (GSH-Px), malondialdehyde (MDA), superoxide dismutase (SOD), and total antioxidant capacity (T-AOC) in the intestines and muscles of adult zebrafish were evaluated as indicators of oxidative stress. As shown by the results, the antioxidant capability of zebrafish was significantly compromised upon TWTP effluent exposure, as shown by significant inhibition of GSH (*p* < 0.05) in gut tissue at 4 months, and significant reduction of MDA and SOD activities (*p* < 0.01) in muscle tissue at the end of 1 month in the treatment group (compared with those of the control). Moreover, although no statistical significances were reached in the concentrations of CAT, GSH-Px, MDA, SOD, and T-AOC in the gut tissue and that of CAT, GSH, GSH-Px, and T-AOC in the muscle tissue in case of the treatment and control groups, there was markedly changes in the concentrations of these substances in the tissues of adult zebrafish treated with TWTP effluents.

Oxidative stress is caused by an imbalance between the generation and removal of ROS (Jia et al., 2021; Yang et al., 2021). Fish frequently face challenges of toxic and harmful pollutants in aquatic environments, leading to environmental oxidative stress. Fish use antioxidant defense systems to scavenge reactive oxygen species (ROS). For the comprehensive toxicity of TWTP effluents exposure, increases of ROS in zebrafish can produce oxidative stress. Elevated ROS impairs mitochondrial function and leads to excessive opening of mitochondrial channels (Hussain et al., 2017; Chen L.J. et al., 2021). Excessive ⋅O_2_^–^ efflux from mitochondria endangers the health of zebrafish. In the antioxidant system, SOD converts ⋅O_2_^–^ to H_2_O_2_, and the resulting H_2_O_2_ can be reduced by CAT and GSH (Weisiger and Fridovich, 1973; Mittler, 2017). The major antioxidant enzymes (including CAT, GSH, and SOD mentioned above) represent the first line of the antioxidant defense system against ROS.

In our study, significantly lower GSH was found in the treatment group than control. It can be explained by the fact that intracellular antioxidant enzymes require GSH to eliminate their antioxidant effects, thus depleting a large amount of GSH (Liu et al., 2008; Massarsky et al., 2017). The same phenomenon has been observed in zebrafish exposed to toxic heavy metals (Adeyemi et al., 2015; Jin et al., 2015).

Malondialdehyde is a lipid peroxidation product and known as an essential cofactor of GSH-Px and SOD and plays a significant antioxidative role by binding to the active site of glutathione peroxidase. Increased MDA levels induce lipid peroxidation, resulting in cell toxicity. Thus MDA is considered as a late biomarker of oxidative stress and cellular damage (Muthulakshmi et al., 2018). The level of MDA is positively correlated with the degree of damage to the organism. In this study, increase in MDA (4 months) and decrease in CAT and SOD of gut tissue in the treatment group indicated that the treatment increased the extent of injury to the organism.

Superoxide dismutase catalyzes the dismutation of the highly reactive O^2–^ to O_2_ and less reactive H_2_O_2_ and is known as an important component of the antioxidant defense system of fish (Wang X.L. et al., 2021), which is depleted to mitigate the damaging effects of ⋅O_2_^–^. In this study, SOD had decreased significantly due to TWTP effluent exposure at the end of 1 and 4 months, suggesting reduced antioxidant capability in zebrafish, which was in accordance with the chronic effects of wastewater-borne heavy metal and titanium dioxide nanoparticles on rainbow trout (Zeumer et al., 2020). Similar chronic toxicity was also suggested by the relatively lower CAT, GSH, and T-AOC levels in the treatment group.

By assessing sensitive biomarkers after chronic exposure, it was found that antioxidant capability of zebrafish is significantly compromised upon TWTP effluent exposure, which suggested that TWTP effluent exposure causes alterations in the activities of vital antioxidant enzymes in the intestine and muscle of adult male zebrafish.

### Microbial Richness and Diversity in Zebrafish Intestines and Water

After separating the low-quality sequence reads, a total of 496,984 usable reads (ranging from 29,511 to 102,030 per sample) were obtained from the 12 samples. The rarefaction curves appear to reach the saturation plateau (Supplementary Figure 2), indicating that the analysis covered most microbial diversity. Sequence composition analyses revealed 1,276.33 ± 138.02 OTUs from the gut of the control group and 1,154.33 ± 44.64 OTUs from the treatment group. The OTUs of the water microbiota in the control group were higher than those in the treatment group. A similar reduction in Chao1 values was also observed in the present study. Although there were no significant differences, the results of OTUs and Chao1 suggested that the microbiota of zebrafish gut and water in the control group had higher microbial community richness. Therefore, TWTP effluents reduced the gut microbial richness in zebrafish.

The alpha diversity of gut and water microbiota was determined using Shannon and Simpson indices, as shown in Table 1. Environmental stress has been shown to reduce microbial community diversity (Ahmed et al., 2020; Li N. et al., 2021). In this study, although no significant difference was observed, the results of diversity indices (Shannon and Simpson) in the treatment group displayed decreased richness compared with the control group. These findings suggest that the diversity of the gut microbial community is reduced in zebrafish following TWTP effluent exposure. Moreover, Good’s coverage values for all groups were relatively high, suggesting that undetected diversity was negligible.

**TABLE 1:** Species richness and diversity of the microbial communities in zebrafish gut and water exposed to TWTP effluents. Values represents the mean ± SD^a^.

Samples	Treatment	Observed OTUs	Chao 1^b^	Shannon^c^	Simpson^c^	Good’s coverage^d^	Phylogenetic diversity^e^
Zebrafish gut	CGM	1,276.33 ± 138.02	2,030.56 ± 207.72	5.63 ± 0.36	0.91 ± 0.02	0.981 ± 0.002	87.48 ± 8.83
	TGM	1,154.33 ± 44.64	1,880.76 ± 120.04	5.45 ± 0.47	0.89 ± 0.04	0.983 ± 0.001	79.23 ± 2.60
Water	CWM	1,042.00 ± 29.44	1,735.59 ± 81.24	5.19 ± 0.47	0.90 ± 0.04	0.984 ± 0.001	66.70 ± 2.46
	TWM	940.33 ± 195.11	1,606.83 ± 282.00	4.86 ± 1.76	0.83 ± 0.18	0.986 ± 0.002	60.11 ± 8.59

*^a^Values represents the mean ± SD (n = 3).*

*^b^Indicatives of bacterial community richness.*

*^c^Indicatives of bacterial community diversity.*

*^d^Indicatives of bacterial sequencing coverage.*

*^e^Indicatives of bacterial phylogenetic diversity.*

### Microbial Composition, Community Structure of Zebrafish Intestines and Water Samples

Under the stress of environmental pollutants, several abnormalities generally occur in the intestines, including the induction of oxidative stress and dysbiosis of gut microbiota (Dreier et al., 2021; Han T.X. et al., 2021; Wang M. et al., 2021; Yang et al., 2021). Several studies have demonstrated that pollutants can lead to significant alterations in gut microbiota and dysbiosis (Evariste et al., 2019; Bajaj and Khoruts, 2020; Duan et al., 2020; Jia et al., 2021). However, to our knowledge, no studies so far have investigated the chronic toxic effects of TWTP effluents on the intestinal microbiota of fish.

**FIGURE 3:**
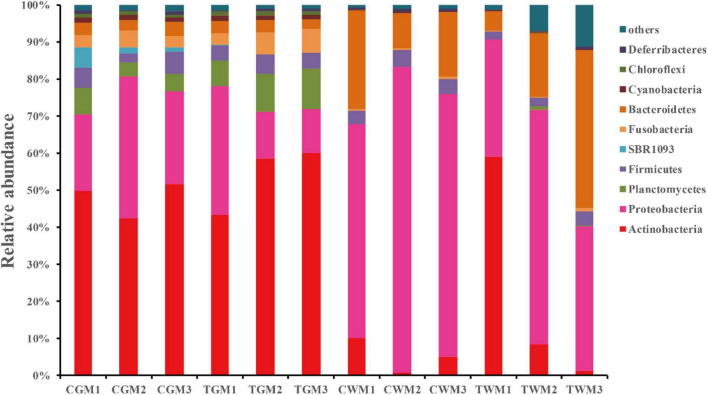
Bacterial composition of the different communities of the dominant phylum present in the zebrafish gut and water among different treatments (4 months exposure). Categories with relative abundance < 1% were clustered into “Other.”

The gut microbial communities of the zebrafish gut and water were analyzed and are presented in Figure 3. A total of 56 phyla were detected in all the samples. In this study, all groups had similar phyla with different relative abundance. At the phylum level, Actinobacteria, Proteobacteria, Planctomycetes, Firmicutes, Fusobacteria, Bacteroidetes, Cyanobacteria, Chloroflexi, and Deferribacteres were the most abundant phyla (>1%) with different relative abundance in all samples. Among these, Actinobacteria, Proteobacteria, Planctomycetes, and Firmicutes were the major phyla in the gut of the control and treatment groups, accounting for 85.85 and 87.59% of the total abundance, respectively, indicating the crucial role of core intestinal microbiota in generalized zebrafish. The results are in accordance with those of previous studies on the gut microbiota of zebrafish (Bao et al., 2020; Kayani et al., 2021). Similar results were found in the water samples of aerated tap water and TWTP effluent exposures of 79.79 and 70.64%, respectively. Proteobacteria of the zebrafish gut in the control group (27.92 ± 9.11) was much higher than that in the treatment group (19.71 ± 13.00). Increased abundance of Planctomycetes was found in the treatment group 9.38 ± 2.13) than in the control (5.25 ± 1.57). Bacteroidetes in the water samples were significantly higher than that in the zebrafish gut. Exposure to TWTP effluents caused considerable variation in the dominant bacterial phyla (Supplementary Table 3). Our results indicated that TWTP effluent exposure markably altered the composition of the gut microbiota at the phylum level.

**FIGURE 4:**
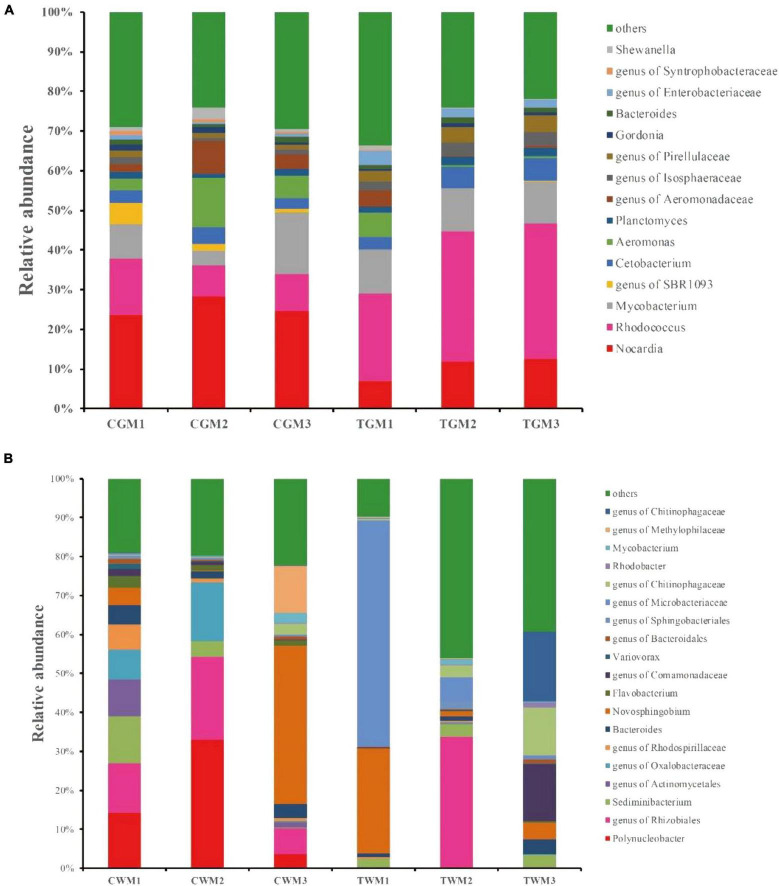
Bacterial composition of the different communities of the dominant genera present in the zebrafish gut **(A)** and water **(B)** among different treatments (4 months exposure). Categories with relative abundance < 1% were clustered into “Other.”

At the genus level, further analysis also showed evident alterations in the gut microbiota composition of zebrafish exposed to TWTP effluents. The most abundant genera in the zebrafish gut were *Nocardia*, *Rhodococcus*, *Mycobacterium*, genus of SBR1093, *Cetobacterium*, and *Aeromonas*. Among these, *Nocardia*, *Rhodococcus*, *Mycobacterium*, and *Cetobacterium* dominated all the samples, making 43.11–63.09% of the total genera, respectively (Figure 4A). *Polynucleobacter*, genus of Rhizobiales, *Sediminibacterium*, genus of Actinomycetales, and genus Oxalobacteraceae were the most abundant genera in the water samples of the control group. The genera Microbacteriaceae, *Novosphingobium*, *Sediminibacterium*, and *Bacteroides* were the most abundant genera in the water of the treatment group (Figure 4B). Among the dominant genera, a significant reduction in *Nocardia* was found in the TWTP effluent-exposed group compared with the control, which was proven to be a pathogenic bacterium (Duperron et al., 2019). Gut microbiota and the host are symbiotic, which work together to maintain the balance of intestinal micro-environment of zebrafish. The exposure of textile effluent pollutant induces changes in the composition of intestinal flora, along with the intestinal immune response. The decrease in the relative abundance of pathogenic bacteria *Nocardia* in this study may have benefited from this process, which merits further study.

The relative abundance of *Rhodococcus* and genera of Isosphaeraceae, Pirellulaceae, and Enterobacteriaceae showed significant increases in the TWTP effluent-exposed group (Supplementary Table 4). Enterobacteriaceae induces inflammation-dependent disruption and disrupts the balance of the original microbial community in the zebrafish gut. Ultimately, it colonizes in the intestinal tract, thereby increasing the risk of intestinal disease in zebrafish (Shealy et al., 2021). *Rhodococcus* are able to fight against toxic substances in the environment and degrade a wide range of organic compounds (Zampolli et al., 2019; Cappelletti et al., 2020). Researchers have observed physiological responses in *Rhodococcus* strains to adapt environmental stimuli, in order to generate greater resistance against exogenous harmful factors (Martínková et al., 2009). *Rhodococcus erythropolis* also adjusts the composition of the bacterial and phospholipid membranes so that the cellular osmotic pressure is appropriate and protects the cell from osmotic stress (Henson et al., 2018).

**FIGURE 5:**
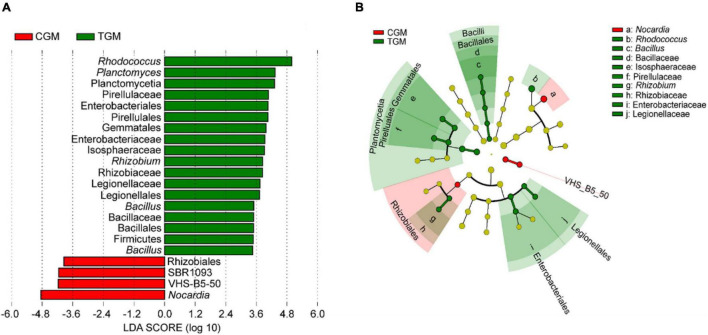
Key phylotypes of the zebrafish gut microbiota responding to the dietary exposure to TWTP effluents, as identified using linear discriminant analysis (LDA) effect size (LEfSe): LDA scores of the differentially abundant taxa of the TWTP effluent exposure group compared with the control group (Note: the higher the LDA score, the greater the contribution to the magnitude of the variation) **(A)**. Cladograms based on the LEfSe analysis for the TWTP effluent groups compared with the control group (red and green circles mean differences in the relative abundance; yellow circles mean non-significant differences) **(B)**.

Linear discriminant analysis effect size (LEfSe) analyses were employed to determine enriched biomarker taxa following TWTP effluent exposure. Twenty-two significantly different taxa at different taxonomic levels between the control and treatment groups were explored using the LEfSe algorithm (Figure 5A). Compared to the control group, the TWTP effluent-exposed group reduced *Nocardia* (Phylum Actinobacteria). As shown in Figure 5B, the taxonomic branches of Bacillus-Bacillaceae-Bacillales in the TWTP effluent-exposed group were underrepresented compared to those in the control group, suggesting that they might serve as potential biomarkers for TWTP effluent exposure in future research.

**FIGURE 6:**
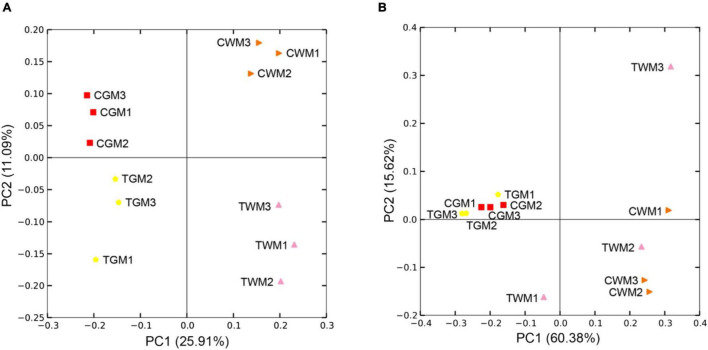
Principal coordinates analysis (PCoA) of the unweighted **(A)** and weighted **(B)** UniFrac scores of the microbial communities. Unweighted principal components (PCs) 1 and 2 explain 25.91 and 11.09% of the variance, while 60.38 and 15.62% based on weighted principal components, respectively.

Based on the PCoA analysis of unweighted and weighted UniFrac distances, the microbial communities of the gut samples were categorized into four clear groups. Visible different microbial community structures were found between the control group and the TWTP effluent-exposed group, whatever in the gut of zebrafish or in the aquatic water (Figure 6). The UPGMA hierarchical cluster analysis also confirmed these results (Supplementary Figure 3). These findings indicate that chronic exposure to TWTP alters the gut microbiota structure of adult zebrafish. Previous studies have shown that imbalances in pathogen resistance, nutrient digestion, and immune modulation may occur due to gut microbiota disorders triggered by environmental pollutants (Evariste et al., 2019; Jia et al., 2021; Li N. et al., 2021; Li X.D. et al., 2021). Thus, our findings highlight the hazards of chronic TWTP effluent exposure affecting the intestinal microbiota that might cause host health in zebrafish.

Previous reports have shown that gut microbes synthesize many essential vitamins, non-essential amino acids, and other substances for the development of the host immune system (Sudo et al., 2004). Probiotics, such as *Lactobacillus*, *Akkermansia*, and *Lactococcus*, produce exopolysaccharides, enhance intestinal barrier function, and maintain the balance of the intestinal microenvironment and modulate host immunity (Newaj-Fyzul et al., 2014; EI-Saadony et al., 2021). The results revealed slight differences in the relative abundance of *Lactobacillus*, *Akkermansia*, and *Lactococcus* in zebrafish guts chronically exposed to TWTP effluents (Supplementary Table 5). A possible reason might be that the members of the probiotic genus could mitigate gut inflammation and sustain barrier function by chronic TWTP effluent exposure (Belzer and de Vos, 2012; Lam et al., 2012), which requires further investigation. At the end of the experiment, a slight increase in the opportunistic pathogens, such as *Mycoplasma*, *Stenotrophomonas*, and *Vibrio*, were also detected. In summary, the diversity, abundance, and relative abundance of zebrafish gut microbiota has become an important indicator of host and environmental health (Nag et al., 2018). Changes in the intestinal microbial community were associated with a reduction in growth performance, antioxidant enzyme activities, and variations in hematobiochemical parameters. Therefore, the chronic toxic effects of TWTP effluents on pathogen increases deserve further attention.

## Conclusion

In conclusion, this study demonstrated that chronic exposure to TWTP effluents can lead to growth inhibition, oxidative stress impairment of the intestine and muscle, and intestinal microbial community alterations in adult zebrafish. However, it should be noted that comprehensive chronic toxic effects were observed in this study. However, the precise toxic substances and mechanisms of toxicity are still unknown and merit further investigation. Further extensive studies focused on screening for high-risk toxic substances, and toxicological mechanisms would provide more insights into the environmental risks of TWTP effluents under real conditions.

## Data Availability Statement

The original contributions presented in the study are publicly available. This data can be found here: https://www.ncbi.nlm.nih.gov/bioproject/PRJNA767223/ and https://www.ncbi.nlm.nih.gov/bioproject/PRJNA767242/.

## Author Contributions

CW and SL designed the study and wrote the manuscript. ZY and YS conducted the experiments and collected the samples. XY performed the microbial diversity analyses. RL contributes to the reviewing and editing of the manuscript. All authors have read and approved the final version of the manuscript.

## Conflict of Interest

The authors declare that the research was conducted in the absence of any commercial or financial relationships that could be construed as a potential conflict of interest.

## Publisher’s Note

All claims expressed in this article are solely those of the authors and do not necessarily represent those of their affiliated organizations, or those of the publisher, the editors and the reviewers. Any product that may be evaluated in this article, or claim that may be made by its manufacturer, is not guaranteed or endorsed by the publisher.
