# Supplementary material for: Effect of Chronic Exposure to Textile Wastewater Treatment Plant Effluents on Growth Performance, Oxidative Stress, and Intestinal Microbiota in Adult Zebrafish (Danio rerio)
Source: Front Microbiol. 2021 Nov 25;12:782611. doi: 10.3389/fmicb.2021.782611 (PMC8656261; doi:10.3389/fmicb.2021.782611)
Supplement: Supplementary file 1 [file Data_Sheet_1.docx]

**Effect of chronic exposure to textile wastewater treatment plant effluents on growth performance, oxidative stress, and intestinal microbiota in adult zebrafish (*Danio rerio*)**

Chun Wang ^1, 2^, Zixi Yuan ^1, 2^, Yingxue Sun ^1, 2^, Xiaolong Yao ^1, 2^, Ruixuan Li ^1, 2^, Shuangshuang Li ^3*^

^1^ School of Ecology and Environment, Beijing Technology and Business University, Beijing 100048, China

^2^ State Environmental Protection Key Laboratory of Food Chain Pollution Control, Beijing Technology and Business University, Beijing 100048, China

^3^ College of Energy and Environmental Engineering, Hebei University of Engineering, Handan 056038, China

Corresponding author.

*E-mail address*: [lishuangs2010@163.com](mailto:lishuangs2010@163.com) (S. Li).

**Fig. S1.** Mortality of zebrafish exposed to aerated tap water (control) and TWTP effluents (treatment) for 4 months. Values presented are mean ± SD of 3 individual tanks (n=3). Treatments with different letters are signiﬁcantly different (P < 0.05).


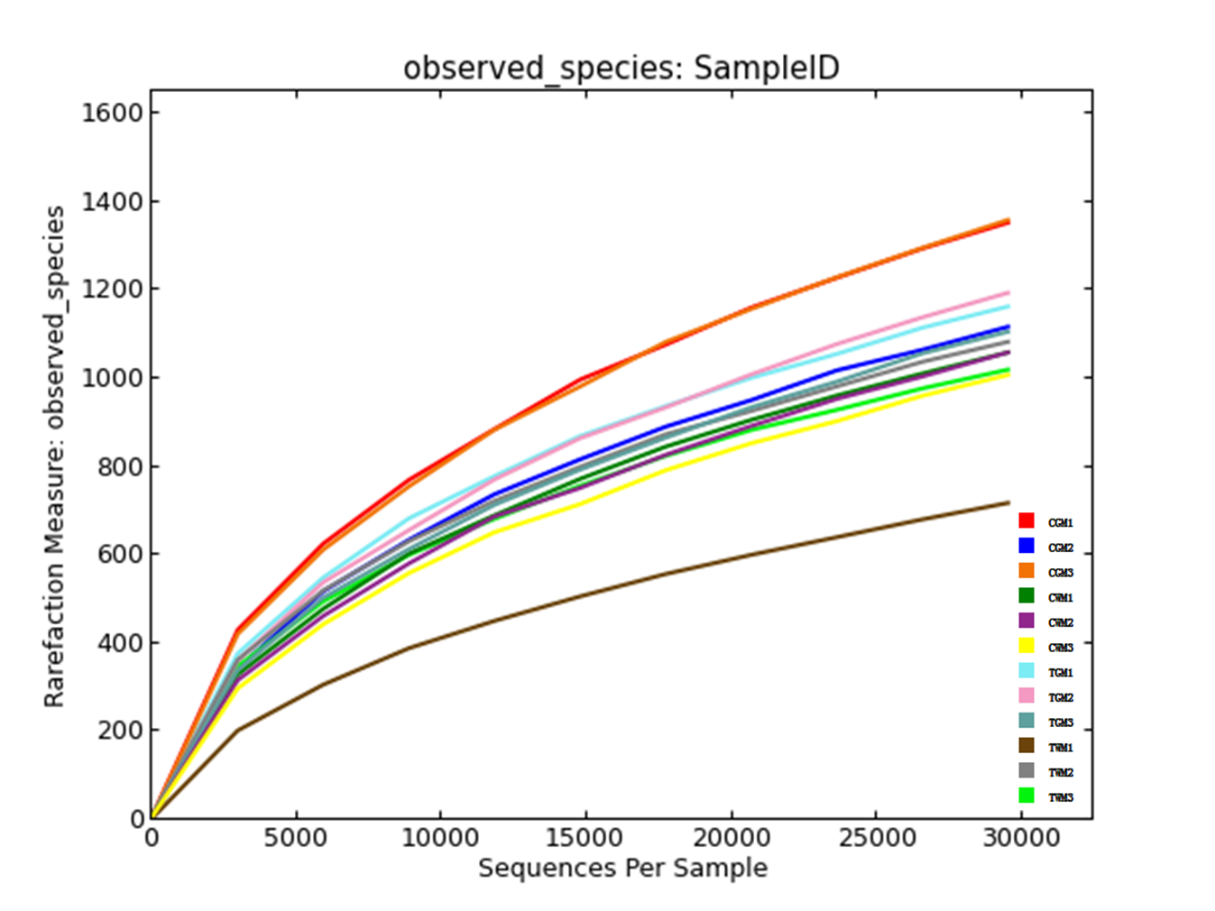


**Fig. S2.** Rarefaction analysis of the different samples. Rarefaction curves of OTUs clustered at 97% similarity. Horizontal axis: the number of valid sequences; vertical axis: the observed number of OTUs. CGM: gut microbiota of control group; TGM: gut microbiota of TWTP effluent exposure group; CWM: water microbiota of control group; TWM: water microbiota of TWTP effluent exposure group.


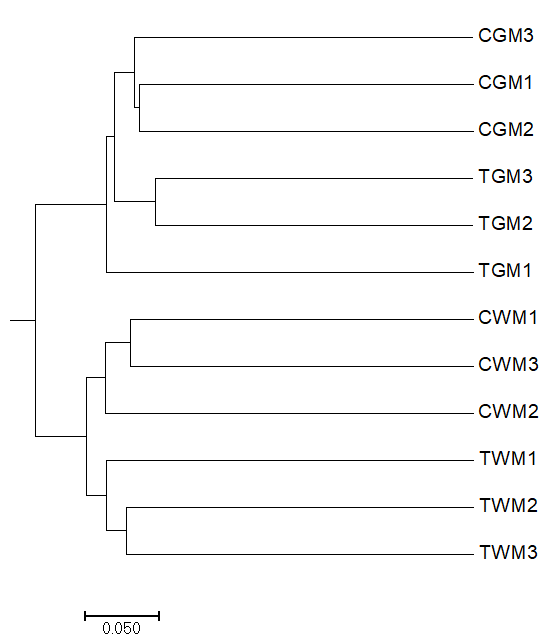


**Fig. S3.** Hierarchical cluster analysis using the UPGMA (unweighted pair group method with arithmetic mean) algorithm generated from taxa tables.

**Table S1.** Primers used for 16S rRNA high-throughout sequencing of V4-V5 hyper-variable region.

| **Primer Name** | **Sequence(5'—3')** | **Reference** |
| --- | --- | --- |
| 515F | GTGYCAGCMGCCGCGGTA | Ye et al., 2014 |
| 909R | CCCGYCAATTCMTTTRAGT |  |

**Table S2**

Growth and condition factor in adult zebraﬁsh after exposure to TWTP effluents.

|  | Weight (g) | Length (cm) | K (%) | HSI (%) |
| --- | --- | --- | --- | --- |
| Control | 0.35 ± 0.03 | 3.44 ± 0.12 | 0.86 ± 0.06 | 1.18 ± 0.15 |
| Treatment | 0.32 ± 0.03** | 3.20 ± 0.18** | 0.98 ± 0.12** | 1.07 ± 0.20* |

K-factor: condition factor calculated as (weight/length^3^) × 100. HSI = liver weight/body weight × 100.

*P < 0.05, **P < 0.01 indicate signiﬁcant difference between exposure group and the control group using one-way analysis of variance (ANOVA) followed by Duncan’s multiple comparisons. Values represent the mean ± SD.

**Table S3.** Relative abundances of most abundant phyla (＞1%) compositions in zebrafish gut and water. Values represents the mean ± SD.

| **Phylum** | **CGM** | **TGM** | **CWM** | **TWM** |
| --- | --- | --- | --- | --- |
| **Actinobacteria** | 48.06±4.92 | 53.99±9.28 | 5.23±4.64 | 22.96±31.46 |
| **Proteobacteria** | 27.92±9.11 | 19.71±13.00 | 70.42±12.37 | 44.60±16.58 |
| **Planctomycetes** | 5.25±1.57 | 9.38±2.13 | 0.08±0.03 | 0.39±0.36 |
| **Firmicutes** | 4.62±1.98 | 4.52±0.60 | 4.07±0.40 | 2.69±0.88 |
| **SBR1093** | 2.71±2.24 | 0.10±0.10 | 0.02±0.01 | 0.02±0.01 |
| **Fusobacteria** | 3.71±0.68 | 5.16±1.73 | 0.47±0.13 | 0.49±0.45 |
| **Bacteroidetes** | 3.26±0.41 | 3.11±0.27 | 17.84±8.52 | 21.68±19.01 |
| **Cyanobacteria** | 1.36±0.09 | 1.26±0.26 | 0.26±0.04 | 0.28±0.11 |
| **Chloroflexi** | 0.92±0.18 | 1.08±0.05 | 0.04±0.02 | 0.03±0.01 |
| **Deferribacteres** | 0.65±0.28 | 0.55±0.13 | 0.71±0.18 | 0.38±0.15 |
| **Others** | 1.56±0.19 | 1.14±0.19 | 0.87±0.09 | 6.47±5.07 |

**Table S4.** Relative abundances of dominant genus compositions (＞1%) in zebrafish gut microbiota. Data with significant differences are shown in bold.

| **Genus** | **Control** | **TWTP effluent exposure** | **P value** |
| --- | --- | --- | --- |
| *Nocardia* | 25.53±2.49 | 10.45±3.14 | **0.003** |
| *Rhodococcus* | 10.48±3.45 | 29.73±6.61 | **0.011** |
| *Mycobacterium* | 9.24±5.88 | 10.80±0.30 | 0.671 |
| *genus of* SBR1093 | 2.71±2.24 | 0.10±0.01 | 0.115 |
| *Cetobacterium* | 3.37±0.73 | 4.70±1.49 | 0.234 |
| *Aeromonas* | 7.01±4.92 | 2.37±3.34 | 0.247 |
| *Planctomyces* | 1.50±0.45 | 1.99±0.52 | 0.283 |
| *genus of* Aeromonadaceae | 4.63±3.30 | 1.61±2.20 | 0.259 |
| *genus of* Isosphaeraceae | 1.24±0.48 | 2.94±0.58 | **0.017** |
| *genus of* Pirellulaceae | 1.40±0.34 | 3.63±0.87 | **0.014** |
| *Gordonia* | 1.14±0.54 | 0.69±0.25 | 0.263 |
| *Bacteroides* | 1.18±0.37 | 1.19±0.32 | 0.958 |
| *genus of* Enterobacteriaceae | 0.84±0.26 | 2.64±1.06 | **0.046** |
| *genus of* Syntrophobacteraceae | 0.61±0.36 | 0.15±0.06 | 0.099 |
| *Shewanella* | 1.64±1.31 | 0.38±0.56 | 0.202 |
| *others* | 27.49±3.06 | 26.60±6.22 | 0.183 |

Note: Values are mean ± SD (n=3).

Signiﬁcantly different was set as P < 0.05.

**Table S5.** The probiotics and potential pathogens in the intestinal of zebrafish following TWTP effluent exposure.

| **Category** | **Genus** | **Control** | **TWTP effluent exposure** | **P value** |
| --- | --- | --- | --- | --- |
| Probiotics | *Lactobacillus* | 0.11±0.04 | 0.12±0.04 | 0.88 |
|  | *Akkermansia* | 0.04±0.01 | 0.02±0.01 | 0.74 |
|  | *Lactococcus* | 0.02±0.02 | 0.02±0.01 | 0.92 |
|  |  |  |  |  |
| Pathogens | *Mycoplasma* | 0.18±0.06 | 0.21±0.07 | 0.63 |
|  | *Stenotrophomonas* | 0.19±0.03 | 0.23±0.11 | 0.55 |
|  | *Vibrio* | 0.01±0.01 | 0.03±0.01 | 0.14 |
|  | *Janthinobacterium* | 0.02±0.00 | 0.01±0.01 | 0.09 |
|  | *Burkholderia* | 0.01±0.01 | 0.01±0.01 | 0.83 |

Note: Values are mean ± SD (n=3).

Signiﬁcantly different was set as P < 0.05.
